# Effects of Nitrogen Fertilization on Synthesis of Primary and Secondary Metabolites in Three Varieties of Kacip Fatimah (*Labisia Pumila* Blume)

**DOI:** 10.3390/ijms12085238

**Published:** 2011-08-16

**Authors:** Mohd Hafiz Ibrahim, Hawa Z.E. Jaafar, Asmah Rahmat, Zaharah Abdul Rahman

**Affiliations:** 1 Department of Crop Science, Faculty of Agriculture, University Putra Malaysia, Serdang, Selangor 43400, Malaysia; E-Mail: mhafizphd@gmail.com; 2 Department of Nutrition & Dietetics, Faculty of Medicine & Health Sciences, University Putra Malaysia, Serdang, Selangor 43400, Malaysia; E-Mail: asmah@medic.upm.edu.my; 3 Department of Land Management, Faculty of Agriculture, University Putra Malaysia, Serdang, Selangor 43400, Malaysia; E-Mail: zaharah@agri.upm.edu.my

**Keywords:** total phenolics, total flavonoids, total non structurable carbohydrate, net assimilation rate, phenyl alanine lyase activity

## Abstract

A split plot 3 by 4 experiment was designed to examine the impact of 15-week variable levels of nitrogen fertilization (0, 90, 180 and 270 kg N/ha) on the characteristics of total flavonoids (TF), total phenolics (TP), total non structurable carbohydrate (TNC), net assimilation rate, leaf chlorophyll content, carbon to nitrogen ratio (C/N), phenyl alanine lyase activity (PAL) and protein content, and their relationships, in three varieties of *Labisia pumila* Blume (*alata*, *pumila* and *lanceolata*). The treatment effects were solely contributed by nitrogen application; there was neither varietal nor interaction effect observed. As nitrogen levels increased from 0 to 270 kg N/ha, the production of TNC was found to decrease steadily. Production of TF and TP reached their peaks under 0 followed by 90, 180 and 270 kg N/ha treatment. However, net assimilation rate was enhanced as nitrogen fertilization increased from 0 to 270 kg N/ha. The increase in production of TP and TF under low nitrogen levels (0 and 90 kg N/ha) was found to be correlated with enhanced PAL activity. The enhancement in PAL activity was followed by reduction in production of soluble protein under low nitrogen fertilization indicating more availability of amino acid phenyl alanine (phe) under low nitrogen content that stimulate the production of carbon based secondary metabolites (CBSM). The latter was manifested by high C/N ratio in *L. pumila* plants.

## 1. Introduction

*Labisia pumila* (Myrsinaceae), also known as Kacip Fatimah, is an herb that has been widely applied as a decoction in South East Asian communities for a variety of illnesses and also used as health supplements [1]. This plant has a long history of use by Malaysian women to ease childbirth as well as to treat post partum illnesses and, therefore, is popularly known as the “queen of plants” of all Malaysian herbs [2]. Stone [3] categorized this herb in Malaysia into three varieties namely *L. pumila* var. *alata*, *L. pumila* var. *pumila* and *L. pumila* var. *lanceolata*. Each of the varieties has a different use [4]. Traditionally, *L. pumila* extract is prepared by boiling the roots, leaves or the whole plant in water and the extract is taken orally and used to accelerate labor, shrink the uterus, improve the menstrual cycle and for weight loss [5].The antioxidant activity of the aqueous *L. pumila* extract has been reported as providing significant protection to human dermal fibroblasts and from cell damage caused by UV irradiation [6], most likely due to the presence of secondary metabolites, *i.e*. flavonoids and phenolics [7].

Flavonoids and other phenolic acids are believed to be responsible for the wide spectrum of pharmacological activities attributed to the herb [8]. Flavonoids are polyphenolic compounds that contain a C15 flavone skeleton (diphenylpropane). They consist of flavones, flavonols, flavanols, flavanone and flavanonols, and represent the majority of plant secondary metabolites. Flavonoids are thought to play a role in protection of plants from microbial and insect attack. Moreover, flavonoids have remarkable health promoting effects, such as anti-inflammatory [9], anti-microbial [10], antioxidant [11], anti cancer activity [12] as well as the prevention of osteoporosis [13]. Besides flavonoids, phenolic acids including gallic acid, benzoic acids and cinnamic acids, constitute another major group of plant secondary metabolites. Nowadays, phenolic acids receive considerable attention because of their protective role against cancer and heart disease. This role may be attributed to their antioxidant activity, which was reported to be higher than vitamins C and E, against reactive oxygen species [14].

The concentration of total flavonoids and phenolics metabolites was found to be influenced by environmental conditions such as light intensity, carbon dioxide levels, temperature, fertilization, and biotic and abiotic factors, which can change the concentration of these active constituents [15]. Nitrogen (N) is one of the main nutrients required for plant growth and is, therefore, applied to crops in substantial amounts to ensure big yields. Nitrogen fertilizer was often used in excess in the past; as a consequence, soil and water were subject to heavy pollution [16]. Provision of nitrogen (N), either from organic or mineral fertilizers, has far-reaching consequences for the performance of plants at the biochemical, ecophysiological and ecosystem level [17]. Nitrogen strongly affects the use of environmental resources (water, light), in part because its metabolites (amino acids) and their derivatives (enzymes and co-factors) are of pivotal importance for plant growth and physiology. Environmental conditions, such as fertility of the habitat, can affect the concentration of secondary compounds [18].

Nitrogen availability is thought to effect the concentrations of secondary compounds in a predictable manner. The plant growing in nitrogen-poor condition is thought to contain more secondary metabolites compounds than plants growing in a nitrogen-rich environment. According to the carbon/nutrient balance hypothesis [19], when nitrogen availability in the soil is low, the low resource availability limits the growth of the plants more than the photosynthesis, and plant allocates the extra carbon that cannot be used for growth to the production of carbon based secondary metabolites (CBSM). Based on the growth differentiation balance hypothesis [20], nitrogen availability is not the only environmental factor that influences the secondary metabolites production in a predictable way, but the trade-off occurs between all growth and differentiation processes.

Phenylalanine is a precursor of flavonoids and phenolics synthesis. It is assumed that biosynthesis of polyphenolic compounds may compete with protein synthesis for phe (phenyl alanine) and that secondary metabolites synthesis may be inhibited because of limiting availability under conditions of rapid incorporation into protein in the protein competition model [21]. There is often a positive relationship between activity of PAL (phenyl alanine lyase), the key enzyme of the phenylpropanoid pathway, and accumulation of carbon based secondary metabolite compounds (CBSM) in plant species [22,23]. Increased activity of PAL is usually observed in nitrogen deficient plants [24], and it has been suggested that nitrogen deficient plants increase the availability of ammonia by enhancing PAL activity, leading to accumulation of polyphenolic compounds [21]. Strissel *et al.* [25] also observed down-regulated PAL activity and reduced flavonoids accumulation in young apple leaves supplied with high nitrogen fertilization. Further, N has been shown to modulate global gene expression concerning primary and secondary metabolism [26].

Plants fertilized with high nitrogen levels tend to increase their photosynthesis and enhance biomass [27]. Many studies have investigated the effects of high nitrogen fertilization on the vegetative and plant primary metabolism but relatively few studies have investigated the response of plant CBSM to increasing nitrogen fertilization, particularly on the medicinal value of local Malaysian herb *Labisia pumila*. The objective of this study was to examine the effects of different nitrogen levels on photosynthesis rate, C/N ratio, chlorophyll content, primary (total non structural carbohydrate), secondary (total flavonoids and total phenolics) metabolites, soluble protein and PAL activity in three varieties of *L. pumila*. Relationships among the parameters were also determined to characterize their cross involvement.

## 2. Results and Discussion

### 2.1. Total Phenolics and Total Flavonoids Profiling

Nitrogen levels had a significant (*P* ≤ 0.05) impact on the production of total phenolics and flavonoids (Table 1). There were no varieties or interaction effects observed. As less nitrogen was provided from 270 to 0 kg N/ha, the more TP and TF were produced. *Labisia pumila* Blume accumulated more of their secondary metabolites in the leaves, followed by the stems and then only in the roots. Total flavonoids decreased in the leaves with increasing nitrogen fertilization from 90 to 180 and then 270 kg N/ha by 42, 43 and 57%, respectively, as compared to non N fertilization. Similar trend was observed in total phenolics of the leaves where lowest production (0.427 mg gallic acid/g dry weight) was registered at 270 kg N/ha compared to 0 kg N/ha (1.01 mg gallic acid/g dry weight). The increase in total plant flavonoids and phenolics under limited nitrogen fertilization was also reported in previous studies by Koricheva *et al.* [28] and Felgines *et al.* [29]. The increase in plant secondary metabolites under limited nitrogen fertilization might be due to improved production of total non structural carbohydrates (TNC) as exhibited by the significantly high value of correlation coefficient (*R*^2^ = 0.72; *P* ≤ 0.05) presented in Table 2. At the same time, it was also observed that the increase in sucrose content might exert more influence in the up regulation of secondary metabolites compared to starch content, with the latter registering the lowest correlation coefficients with total flavonoids and phenolics. This data implies that increase in sucrose content could be a possible explanation to increased production of total flavonoids and phenolics in the present study. Similar conclusion was also derived by the findings of Guo *et al.* [30] and Amin *et al.* [31] who proposed that increase in the production of plant secondary metabolites in their studies was due to increase in the production of sucrose as observed in the brocolli and onion, respectively.

### 2.2. Total Soluble Sugar, Starch and Total Non Structurable Carbohydrate (TNC) and Their Profiling

The profiling of carbohydrates was influenced by the nitrogen levels applied to *L. pumila* (*P* ≤ 0.05). There was neither varietal nor interaction effects observed in the study. The accumulation of carbohydrates in different parts of the plant followed a descending order of leaf > stem > root. As the level of nitrogen fertilization increased, the concentration of sucrose, starch and TNC decreased (Table 3). The concentration of sucrose and starch registered the lowest values at 270 kg N/ha compared to other treatments. In the leaves of plants applied with 270 kg N/ha (19.80 mg sucrose/g dry weight), 180 (32.64 mg sucrose/g dry weight) and 90 kg N/ha (35.45 mg sucrose/g dry weight) were found to produce less sucrose than with 0 kg N/ha, which produced 40.70 mg sucrose/g dry weight. The starch content in the leaves of 270 kg N/ha treated plants were statistically lower than other nitrogen fertilization treatments. The starch contents in the leaves of 0, 90 and 180 kg N/ha treated plants in the current work registered higher values of 90.41, 77.18 and 62.22 mg glucose/g dry weight, respectively, in comparison with only 49.66 mg glucose/g dry weight for 270 kg N/ha treated plants. In all plant parts of *L. pumila*, the increase in starch content was larger than the increase in sugar concentration with decreasing N fertilization [31] suggesting that the low N-fertilization was able to enhance soluble sugar and starch contents, which had simultaneously enhanced the TNC. Similar observation was demonstrated by other researchers [32–34]. The accumulation of TNC in low nitrogen-fertilized plant might be due to reduction in sink size of the plant when nitrogen is limited; hence, reducing the translocation of carbohydrates to other plant parts [35].

When sink strength was reduced under low nitrogen fertilization, the extra carbohydrates accumulated in *L. pumila* plants might be channeled for the production of total phenols and flavonoids, thus, explaining the reason why the production of secondary metabolites was enhanced in low nitrogen fertilization (0–90 kg N/ha). As explained by Tognetti and Johnson [36], it is possible that when photosynthetic performance is suppressed under insufficient nitrogen supply, recycling of the enzymatic nitrogen required for secondary metabolism may occur resulting in possible increase in secondary metabolites (phenolics and flavonoids).

### 2.3. Net Photosynthesis Rate

The photosynthesis rate was influenced by nitrogen levels applied. Both differences in varieties and their interactions with N levels did not exert any effect on net photosynthesis. As nitrogen levels increased in an ascending order of 0 > 90 > 180 > 270 kg N/ha, the net photosynthesis rate simultaneously increased steadily (Table 4). The highest photosynthesis rate was obtained in *L. pumila* fertilized with 270 kg N ha^−1^ (10.71 μmol/m^2^/s) followed by 180 kg N/ha (7.31 μmol/m^2^/s), 90 kg N/ha (4.21 μmol/m^2^/s) and the lowest with 0 kg N/ha (2.11 μmol/m^2^/s). Results showed the importance of increasing nitrogen fertilization in further enhancing leaf gas exchange of *L. pumila* plants. However, the photosynthetic rate in the current study was also found to establish a significant negative relationship with the production of secondary metabolites implying that under limitation of nitrogen resource, the stimulation of total flavonoids and phenolics might occur as signified by the low leaf gas exchange properties [37]. Fan *et al.* [38] extended a possible explanation to this phenomenon that a decrease in the photosynthetic rate could have enhanced the shikimic acid and penthose phosphate pathway activities and in turn could have enhanced the production of plant secondary metabolites. The same negative relationship between photosynthesis and production of secondary metabolites was also observed by Ali and Ashraf [39] and Hura *et al.* [40].

### 2.4. Leaf Nitrogen and C/N Ratio

The intensification of N fertilization significantly improved leaf nitrogen content (*P* ≤ 0.05). As nitrogen levels increased from 0 to 270 kg N/ha, leaf tissue nitrogen also increased considerably (Table 4). The leaf nitrogen level was significantly higher in the 270 kg N/ha than in all nitrogen treatments exhibiting an increase in foliar nitrogen content by 38% higher than the average of the other three levels of nitrogen fertilization. The increase in leaf tissue nitrogen might result from intensification of nitrate content in the leaf [41]. Subsequently, the increase in leaf nitrogen content had lead to reduced plant C/N ratio under high N fertilization. The C/N ratio was higher under 0 kg N/ha than under the 90, 180 and 270 kg N/ha treatments by respective of 18%, 38% and 62%. A similar increase in C/N ratio of plants fertilized with low nitrogen was also observed by Anderson *et al.* [42]. The high C/N ratio had a significant positive relationship (*P* ≤ 0.01) with total flavonoids (*R*^2^ = 0.87) and total phenolics compound (*R*^2^ = 0.81; Table 2) signifying a good direct association between the C/N ratio and plant secondary metabolites. Winger *et al.* [43] attributed the increase in C/N ratio to increase in carbohydrate accumulation. In the present study, the increase in C/N ratio was found to have significant positive correlationship with total flavonoids and total phenolics, suggesting that increase in the C/N ratio would have enhanced synthesis of plant secondary metabolites, especially the flavonoids and phenolics, in *L. pumila* [44].

### 2.5. Chlorophyll Content

The production of chlorophyll content was influenced by the application of nitrogen (*P* ≤ 0.01). As the levels of N fertilization increased from 0 to 270 kg N/ha, chlorophyll a, b and total chlorophyll a + b were also improved (Table 4). The increase in chlorophyll content with increasing nitrogen has been reported by Sanewaara *et al.* [45]. It was found from the correlation (Table 2) that chlorophyll a, b and total (a + b) were significantly (*P* ≤ 0.01) and negatively related to the production of secondary metabolites. The negative relationship between chlorophyll content and secondary metabolites production fits well to protein competition model (PCM) proposed by Jones and Hartley [46] that the secondary metabolites content is controlled by the competition between protein and secondary metabolites biosynthesis pathway and the metabolites regulation. The negative relationship between the secondary metabolites and chlorophyll is a sign of gradual switch-off of investment from protein to polyphenols production [47].

### 2.6. Phenyl-Alanine-Lyase Activity (PAL)

The PAL activity in *L. pumila* was influenced by nitrogen levels (*P* ≥ 0.05). There were no varietal effects nor their interaction with N fertilization was observed; and the results from different varieties are not presented. PAL activity was found to be the highest (26.71 nM transcinnamic/mg protein/h) when there was no nitrogen (0 kg N/ha) being applied, and the lowest activity was demonstrated at 270 kg/N ha which registered a value of 7.68 nM transcinnamic/mg protein/h (Table 5). The increase in the production of secondary metabolites in the present work could be related to the increase in PAL activities under low nitrogen levels. Correlation analysis showed that PAL had established a significant (*P* ≤ 0.05) positive relationship with total phenolics (*R*^2^ = 0.94) and flavonoids (*R*^2^ = 0.92), suggesting an up-regulation of plant secondary metabolites production with increased PAL activity. This is basically due to the fact that PAL is an enzyme, which synthesizes a precursor to total phenolics and flavonoids biosynthesis. The low nitrogen fertilization increased the availability of phenyl alanine (Phe) due to restriction in protein production; hence, more Phe is available for the production of secondary metabolites [46]. The increase in PAL activity under low nitrogen level was also observed by Matros *et al.* [48] and Hartley *et al.* [49]. These results suggested that an up-regulation of plant secondary metabolites production in *L. pumila* under low nitrogen might be due to increase in PAL activity.

### 2.7. Soluble Protein Content

Soluble protein content followed a similar trend as that of the PAL activity where the effect of treatment was solely influenced by nitrogen levels (*P* ≤ 0.05). The soluble protein content increased with increasing nitrogen fertilization levels from 0 to 270 kg N/ha (Table 5). At 270 kg N/ha the soluble protein content recorded a value at 11.79 mg/g fresh weight, which was 29%, 67% and 77% higher than the values obtained at 180, 90 and 0 kg N/ha, respectively (in the respective order of 8.26, 3.87 and 2.67 mg/g fresh weight). Similar result to the present study was also observed by Robredo *et al.* [50] in barley and Gleadow *et al.* [51] in cassava where the highest protein accumulation was dictated by high nitrogen fertilization. Protein content was also found to have a negative relationship with total phenols and flavonoids (*R*^2^ = −0.87; R^2^ = −0.78; *P* ≤ 0.05), which indicated the occurrence of an up-regulation of plant secondary metabolites when protein content was reduced [47]. The present study also demonstrated that a decrease in protein production under low nitrogen levels might decrease the use of Phe for protein synthesis which is necessary in the biosynthesis of plant secondary metabolites [52]. This explains why increase in secondary metabolites might be up-regulated under low nitrogen condition.

## 3. Experimental Section

### 3.1. Experimental Location, Plant Materials and Treatments

The experiment was carried out in growth houses at Field 2, Faculty of Agriculture Glasshouse Complex, Universiti Putra Malaysia (longitude 101°44′N and latitude 2°58′S, 68 m above sea level) with a mean atmospheric pressure of 1.013 kPa. Three-month old *L. pumila* seedlings of var. *alata*, var. *pumila* and var. *lanceolata* were left for a month to acclimatize in a nursery until ready for the treatments. When the seedlings had reached 4 months of age and they were fertilized with four rates of nitrogen, viz. 0, 90, 180 and 270 kg N/ha, applied in the form of urea. The seedlings were planted in soilless medium containing coco-peat, burnt paddy husk and well composted chicken manure in 5:5:1 (v/v) ratio in 25 cm diameter polyethylene bags. Day and night temperatures in the greenhouse were maintained at 27–30 °C and 18–21 °C, respectively, and relative humidity from 50 to 60%. All the seedlings were irrigated using overhead mist irrigation given four times a day or when necessary. Each irrigation session lasted for 7 min. Fertilization with nitrogen levels was split into three applications (Table 6). This factorial experiment was arranged in split plot using a randomized complete block design with varieties being the main plot, and nitrogen levels as the sub-plot, and replicated three times. Each treatment consisted of ten seedlings.

### 3.2. Total Phenolics and Total Flavonoids Quantification

The method of extraction and quantification for total phenolics and flavonoids contents followed after Jaafar *et al.* [53]. An amount of ground tissue samples (0.1 g) was extracted with 80% ethanol (10 mL) on an orbital shaker for 120 min at 50 °C. The mixture was subsequently filtered (Whatman™ No.1), and the filtrate was used for the quantification of total phenolics and total flavonoids. Folin-Ciocalteu reagent (diluted 10-fold) was used to determine the total phenolics content of the leaf samples. Two hundred μL of the sample extract was mixed with Follin–Ciocalteau reagent (1.5 mL) and allowed to stand at 22 °C for 5 min before adding NaNO_3_ solution (1.5 mL, 60 g L^−1^). After two hours at 22 °C, absorbance was measured at 725 nm. The results were expressed as mg g^−1^ gallic acid equivalent (mg GAE g^−1^ dry sample). For total flavonoids determination, a sample (1 mL) was mixed with NaNO_3_ (0.3 mL) in a test tube covered with aluminum foil, and left for 5 min. Then 10% AlCl_3_ (0.3 mL) was added followed by addition of 1 M NaOH (2 mL). Later, the absorbance was measured at 510 nm using a spectrophotometer with rutin as a standard (results expressed as mg g^−1^ rutin dry sample).

### 3.3. Total Soluble Sugar Determination

Total soluble sugar was measured spectrophotometrically using the method of Edward [54]. Samples (0.5 g) were placed in 15 mL conical tubes, and distilled water added to make up the volume to 10 mL. The mixture was then vortexed and later incubated for 10 min. Anthrone reagent was prepared using anthrone (Sigma Aldrich, St Louis, MO, USA, 0.1 g) that was dissolved in 95% sulphuric acid (Fisher Scientific, USA 50 mL). Sucrose was used as a standard stock solution to prepare a standard curve for the quantification of sucrose in the sample. The mixed sample of ground dry sample and distilled water was centrifuged at a speed of 3400 rpm for 10 min and then filtered to get the supernatant. A sample (4 mL) was mixed with anthrone reagent (8 mL) and then placed in a water-bath set at 100 °C for 5 min before the sample was measured at an absorbance of 620 nm using a spectrophotometer model UV160U (Shimadzu Scientific, Kyoto, Japan). The total soluble sugar in the sample was expressed as mg sucrose g^−1^ dry sample.

### 3.4. Starch Determination

Starch content was determined spectrophometrically using a method described by Thayumanavam and Sadasivam [55]. In this method, dry sample (about 0.5 g) was homogenized in hot 80% ethanol to remove the sugar. The sample was then centrifuged at 5000 rpm for 5 min and the residue retained. After that, distilled water (5.0 mL) and 52% perchloric acid (6.5 mL) were added to the residue. Then the solution was centrifuged and the supernatant separated and then filtered with Whatman No. 5 filter paper. The processes were repeated until the supernatant was made up to 100 mL. A sample (100 μL) of the supernatant was added to distilled water until the volume became 1 mL in a test tube. After that, anthrone reagent (4 mL, prepared with 95% sulphuric acid) was added to the test tube. The mixed solution was placed in the water bath at 100 °C for eight min and then cooled to room temperature, and then the sample was read at absorbance of 630 nm to determine the sample starch content. Glucose was used as a standard and starch content was expressed as mg glucose equivalent g^−1^ dry sample.

### 3.5. Total Non Structural Carbohydrate (TNC)

The total non structural carbohydrate was calculated as the sum of total soluble sugar and starch content [36].

### 3.6. Photosynthesis Rate

The measurement was obtained from a closed infra-red gas analyzer LICOR 6400 Portable Photosynthesis System (IRGA, Licor Inc. Nebraska, USA). Prior to use, the instrument was warmed for 30 min and calibrated with the ZERO IRGA mode. Two steps are required in the calibration process: first, the initial zeroing process for the built-in flow meter; and second, zeroing process for the infra-red gas analyzer. The measurements used optimal conditions of 400 μmol/mol CO_2_ 30 °C cuvette temperature, 60% relative humidity with air flow rate set at 500 cm^3^/min, and modified cuvette condition of 800 μmol/m^2^/s photosynthetically photon flux density (PPFD). The measurements of gas exchange were carried out between 09:00 to 11:00 a.m. using fully expanded young leaves numbered three and four from plant apex to record net photosynthesis rate (A). The operation was automatic and the data were stored in the LI-6400 console and analyzed by “*Photosyn Assistant*” software (Version 3, Lincoln Inc., USA). Several precautions were taken to avoid errors during measurements. Leaf surfaces were cleaned and dried using tissue paper before enclosed in the leaf cuvette [56].

### 3.7. Total Carbon, Nitrogen and C:N Ratio

Total carbon and C:N ratio were measured by using a CNS 2000 analyzer (Model A Analyst 300, LECO Inc., USA). This was performed by placing 0.05 g of ground leaf sample into the combustion boat. Successively, the combustion boat was transferred to the loader before the sample was burned at 1350 °C to obtain the reading of total carbon and nitrogen content of the samples.

### 3.8. Chlorophyll Content

Total chlorophyll content was measured by method from Ibrahim and Hawa [57] using fresh weight basis. Prior to each destructive harvest each seedling was analyzed for the leaf chlorophyll relative reading (SPAD meter 502, Minolta Inc., USA). The leaves of *L. pumila* with different greenness (yellow, light green and dark green) were selected for analysis and total leaf chlorophyll content was analyzed. For each type of leaf greenness, the relative SPAD value was recorded (five points/leaf) and the same leaves sampled for chlorophyll content determination. Leaf disk 3 mm in diameter was obtained from leaf sample using a hole puncher. For each seedling the measurement was conducted on the youngest fully expanded leaves on each plant, generally the second or third leaf from the tip of the stem was used. The leaf disks were immediately immersed in acetone (20 mL) in an aluminum foil-covered glass bottle for approximately 24 h at 0 °C until all the green colour had bleached out. Finally, the solution (3.5 mL) was transferred to measure at absorbances of 664 and 647 nm using a spectrometer (UV-3101P, Labomed Inc., USA). After that the least squares regression was used to develop predictive relation between SPAD meter readings and pigment concentrations (mg g^−1^ fresh weight) obtained from the chlorophyll destructive analysis.

### 3.9. Phenylalanine-Ammonia-Lyase (PAL)

Phenylalanine-ammonia-lyase (PAL) activity was measured using the method described by Ibrahim and Hawa [58]. The enzyme activity was determined by measuring spectrophotometrically the production of *trans*-cinnamic acid from l-phenylalanine. Enzyme extract (10 μL) was incubated at 40 °C with 12.1 mM L-phenylalanine (90 μL, Sigma) that were prepared in 50 mM Tris-HCl, (pH 8.5). After 15 min of reaction, *trans*-cinnamic acid yield was estimated by measuring increase in the absorbance at 290 nm. Standard curve was prepared by using a *trans*-cinnamic acid standard (Sigma) and the PAL activity was expressed as nM *trans*-cinnamic acid/μg protein/h.

### 3.10. Protein Determination

Protein content was determined using the method of Bradford [59]. In this method, fresh leaf samples (about 2 g) were cut into pieces using scissors and ground in mortar with 0.05 M Tris buffer (1 mL, pH 8.5) and powdered with liquid nitrogen. The homogenate was then centrifuged at 9,000 rpm for 10 min and then stored under refrigeration at 4 °C for 24 h. After the extraction, supernatant from the samples (about 100 μL) was added to Bradford reagent (3 mL, Sigma, prepared using 10 mL of the reagent diluted with 50 mL distilled water) and then incubated for 5 min before being measured at 595 nm with the spectrophotometer. In this method bovine serum (Sigma) was used as a standard to produce calibration curve between actual protein content and spectrophotometer readings. The protein was expressed as mg g protein/fresh weight.

### 3.11. Statistical Analysis

Data were analyzed using analysis of variance using SAS version 17. Mean separation test between treatments was performed using Duncan multiple range test and standard error of differences between means was calculated with the assumption that data were normally distributed and equally replicated.

## 4. Conclusions

This study demonstrated that fertilization with high nitrogen fertilizer can reduce the production of total flavonoids and phenolics in *L. pumila*. The increase in production of CBSM under low nitrogen fertilization was followed by increase in production of TNC. The low N fertilized plants demonstrated negative correlation with total chlorophyll content and registered high C/N ratio. From the present study, it can be deduced that increase in production of CBSM under low nitrogen fertilization in *L. pumila* might be due to increase in availability of phenyl alanine, as shown by high PAL activity due to restriction in protein production under low nitrogen fertilization.

## Figures and Tables

**Table 1.** Accumulation and partitioning of total flavonoids and total phenolics in different plant parts of *Labisia pumila* Blume, as affected by different nitrogen levels.

**Nitrogen**	**Plant parts**	**Total flavonoid(mg rutin equivalent/g dry weight)**	**Total phenolics(mg gallic acid equivalent/g dry weight)**
0 kg N/ha	Leaf	0.713 ± 0.013 ^a^	1.010 ± 0.028 ^a^
	Stem	0.628 ± 0.022 ^a^	0.964 ± 0.029 ^a^
	Root	0.528 ± 0.013 ^b^	0.882 ± 0.039 ^b^
90 kg N/ha	Leaf	0.415 ± 0.022 ^b^	0.987 ± 0.032 ^a^
	Stem	0.378 ± 0.030 ^b^	0.876 ± 0.037 ^b^
	Root	0.298 ± 0.022 ^b^	0.677 ± 0.051 ^c^
180 kg N/ha	Leaf	0.400 ± 0.013 ^c^	0.711 ± 0.021 ^b^
	Stem	0.325 ± 0.010 ^c^	0.613 ± 0.025 ^c^
	Root	0.264 ± 0.015 ^d^	0.434 ± 0.040 ^d^
270 kg N/ha	Leaf	0.301 ± 0.025 ^c^	0.427 ± 0.008 ^d^
	Stem	0.106 ± 0.023 ^d^	0.321 ± 0.011 ^e^
	Root	0.077 ± 0.026 ^d^	0.230 ± 0.028 ^e^

All analyses are mean ± standard error of mean (SEM). *N* = 18. Means not sharing a common alphabet were significantly different at *P* ≤ 0.05.

**Table 2.** Correlations among the measured parameters in the experiment.

**Characteristics**	**1**	**2**	**3**	**4**	**5**	**6**	**7**	**8**	**9**	**10**	**11**	**12**	**13**
1. TF	1.00												
2. TP	0.98 *	1.00											
3. Sucrose	0.89 *	0.76 *	1.00										
4. Starch	0.78 *	0.67	0.76 *	1.00									
5. TNC	0.72 *	0.62 *	0.72 **	0.87 *	1.00								
6. Photosynthesis	−0.87 *	−0.82 **	−0.79 **	−0.88 **	−0.78 *	1.00							
7. N	−0.91 *	−0.89 **	−0.76 *	0.89 *	0.91 **	0.78 *	1.00						
8. C/N	0.87 *	0.81 *	0.87 **	0.93 **	0.79 **	−0.67 *	−0.92 **	1.00					
9. Chl a	−0.78 *	−0.76 **	−0.70 *	−0.78 *	−0.78 **	−0.67 **	0.88 *	−0.87 **	1.00				
10. Chl b	−0.76 *	−0.82 **	−0.73 **	−0.86 **	−0.68	−0.71	0.89 **	−0.85 *	0.76	1.00			
11. Chl a + b	−0.78 *	−0.75 **	−0.76 *	−0.75 *	−0.72 *	−0.74 **	0.90 **	−0.82 **	0.75 *	0.87 *	1.00		
12. PAL	0.92 **	0.94 **	0.78 *	0.56	0.71 *	−0.45	−0.89 *	−0.67	−0.76 *	−0.72 *	−0.56	1.00	
13. Protein	−0.87 *	−0.78 *	−0.72 **	−0.79 *	−0.87 *	0.76 *	0.96 *	−0.76 *	0.87 **	0.83 **	0.87 **	−0.79 *	1.00

* and ** respectively, significant at *P* ≤ 0.05 or *P* ≤ 0.01.

**Table 3.** Accumulation and partitioning of total soluble sugar (TSS), starch and total non structurable carbohydrate (TNC) in different plant parts of *Labisia pumila* Blume as affected by different nitrogen levels.

**Nitrogen levels**	**Plant parts**	**TSS (mg sucrose/g dry weight)**	**Starch (mg glucose/g dry weight)**	**TNC (mg/g dry weight)**
0 kg N/ha	Leaf	40.71 ± 0.50 ^a^	90.41 ± 0.28 ^a^	131.32 ± 3.22 ^a^
	Stem	31.04 ± 0.84 ^c^	87.26 ± 0.29 ^a^	117.21 ± 1.46 ^b^
	Root	35.09 ± 0.50 ^b^	80.22 ± 0.49 ^b^	114.34 ± 3.26 ^b^
90 kg N/ha	Leaf	35.45 ± 0.49 ^b^	77.18 ± 0.62 ^b^	112.17 ± 4.77 ^b^
	Stem	23.26 ± 0.44 ^d^	65.27 ± 0.47 ^c^	88.23 ± 2.22 ^c^
	Root	21.66 ± 0.49 ^d^	60.98 ± 0.51 ^c^	80.21 ± 5.55 ^c^
180 kg N/ha	Leaf	32.64 ± 0.59 ^c^	62.22 ± 0.23 ^c^	83.78 ± 4.67 ^c^
	Stem	26.10 ± 0.99 ^d^	59.03 ± 0.25 ^c^	85.31 ± 4.90 ^c^
	Root	19.86 ± 0.58 ^d^	52.67 ± 0.44 ^d^	71.75 ± 1.90 ^d^
270 kg N/ha	Leaf	19.80 ± 1.16 ^d^	49.66 ± 0.23 ^d^	68.22 ± 5.89 ^d^
	Stem	15.96 ± 0.70 ^e^	32.42 ± 0.21 ^e^	47.31 ± 1.65 ^e^
	Root	9.02 ± 1.17 ^e^	29.20 ± 0.28 ^e^	37.31 ± 9.96 ^e^

All analyses are mean ± standard error of mean (SEM), *N* = 18. Means not sharing a common single alphabet were significantly different at *P* ≤ 0.05.

**Table 4.** The effect of nitrogen levels on photosynthesis, leaf nitrogen, C/N ratio and chlorophyll content in *L. pumila*.

**Parameters**	**0 kg N/ha**	**90 kg N/ha**	**180 kg N/ha**	**270 kg N/ha**
Photosynthesis (μmol/m^2^/s)	2.11 ± 0.25 ^d^	4.21 ± 0.34 ^c^	7.31 ± 0.12 ^b^	10.71 ± 0.43 ^a^
Leaf Nitrogen (%)	0.76 ± 0.12 ^d^	2.44 ± 0.02 ^c^	2.89 ± 0.23 ^b^	3.26 ± 0.43 ^a^
C/N	27.15 ± 0.45 ^a^	22.16 ± 0.45 ^b^	16.73 ± 0.65 ^c^	10.22 ± 0.56 ^d^
Chlorophyll a (mg/g fresh weight)	3.60 ± 0.08 ^d^	4.44 ± 0.09 ^c^	5.42 ± 0.04 ^b^	6.47 ± 0.01 ^a^
Chlorophyll b (mg/g fresh weight)	12.76 ± 0.23 ^d^	14.72 ± 0.34 ^c^	18.23 ± 0.09 ^b^	21.27 ± 0.23 ^a^
T. Chlorophyll (mg/g fresh weight)	15.34 ± 0.34 ^d^	19.21 ± 0.31 ^c^	23.54 ± 0.78 ^b^	27.23 ± 0.55 ^a^

All analyses are mean ± standard error of mean (SEM), *N* = 18. Means not sharing a common single alphabet were significantly different at *P* ≤ 0.05.

**Table 5.** The effect of nitrogen levels on soluble protein and PAL activity in *L. pumila*.

**Nitrogen levels**	**Soluble protein(mg/g fresh weight)**	**PAL (Phenyl-alanine-lyase) activity (nM transcinnamic/mg protein/h)**
0 kg N/ha	2.67 ± 0.24 ^d^	26.71 ± 0.78 ^d^
90 kg N/ha	3.87 ± 0.12 ^c^	21.45 ± 1.21 ^c^
180 kg N/ha	8.26 ± 0.09 ^b^	13.65 ± 0.98 ^b^
270 kg N/ha	11.76 ± 0.78 ^a^	7.68 ± 0.87 ^a^

All analyses are mean ± standard error of mean (SEM), *N* = 18. Means not sharing a common single alphabet were significantly different at *P* ≤ 0.05.

**Table 6.** Nitrogen fertilization levels of *Labisia pumila* Blume during the experiment.

**Nitrogen (kg N/ha) ^1,2^**	**Total nitrogen fertilizer per plant ^3^ (g)**
0	0.00
90	0.36
180	0.72
270	1.08

1 Nitrogen source used was urea (46% N);

2 Every nitrogen treatment receives TSP (Triple super phosphate; 46% P) and MOP (muriate of potash; 60% K) at standard rates of 180 kg N ha^−1^; the nitrogen was split into three fertilization phases, and each phase was about 33.3% of total nitrogen fertilizer;

3 Every nitrogen treatment receives TSP (triple super phosphate; 46% P; 0.72 g per plant) and MOP (60% K; 0.51 g per plant) at standard rates of 180 kg N/ha.
